# Exact firing-rate response of the integrate-and-fire neuron receiving finite amplitude excitatory and inhibitory post-synaptic potentials

**DOI:** 10.1186/1471-2202-12-S1-O18

**Published:** 2011-07-18

**Authors:** Magnus JE Richardson, Rupert Swarbrick

**Affiliations:** 1 Warwick Systems Biology Centre, University of Warwick, CV4 7AL, UK

## 

Neurons in active networks are subject to a fluctuating synaptic drive comprising excitatory and inhibitory post-synaptic potentials arriving at high rates relative to the integration time of the cell. The standard approach for treating stochastic synaptic currents and conductances has been to approximate them by a Gaussian white-noise or coloured-noise process in which the amplitudes of the underlying post-synaptic potentials are considered small. Importantly, this diffusion approximation has allowed for various firing-rate properties of populations of fluctuation-driven neurons to be calculated using Johanessma’s [1] and Ricciardi’s [2] Fokker-Planck framework. This framework in turn has proven to be a powerful theoretical tool for deriving the emergent states of coupled neuronal networks as a function of the properties of constituent neurons. The Gaussian/diffusion model of fluctuating synaptic conductance is in widespread use theoretically, computationally and experimentally and, in many cases, is an excellent approximation to the synaptic drive experienced by neurons in active networks *in vitro* or *in vivo*.

The typical synaptic coupling between neurons in many brain regions, however, is often sufficiently strong such that relatively few synchronous synaptic events are required to bring a neuron from rest to the spiking threshold. For example, pairs of neocortical layer-5 pyramidal cells form synaptic connections with amplitudes in the range 0.5mV-6mV with a mean of 1.3mV [2] and, when correlations are taken into account, near-synchronous pre-synaptic activity will lead to even stronger aggregate EPSPs. Given that the difference between rest and threshold is around 10mV for this class of cell [3] it is likely that finite-amplitude effects missed by the standard Gaussian/diffusion approximation will play an important role in shaping the steady-state and response properties of neurons *in vivo*.

Here we present a significant generalization of the standard Gaussian/diffusion approach to treating synaptic fluctuations by exactly solving the firing-rate response for a neuron receiving finite-amplitude Poissonian excitatory and inhibitory post-synaptic potentials [5]. The mathematical framework involves the solution of a master equation and can be applied to synaptic-amplitude distributions decomposable into combinations of exponential functions -similar to those seen in experiment. It will be shown that this more general mathematical description of the neuronal response to stochastic synaptic dynamics is significantly richer and qualitatively distinct from that predicted by the Gaussian/diffusion approximation. As well as providing analytical solutions for the firing-rate response of the leaky integrate-and-fire model, it will be demonstrated how an efficient numerical scheme can be used to calculate, numerically but exactly, the response properties of non-linear integrate-and-fire neurons and also to better capture the effects of synaptic reversal potentials.
